# Metabolic-Associated Fatty Liver Disease and Cognitive Performance in Type 2 Diabetes: Basal Data from the Phytate, Neurodegeneration and Diabetes (PHYND) Study

**DOI:** 10.3390/biomedicines12091993

**Published:** 2024-09-02

**Authors:** Antelm Pujol, Pilar Sanchis, María I. Tamayo, Samantha Godoy, Paula Calvó, Asier Olmos, Pilar Andrés, Aleksandra Speranskaya, Ana Espino, Ana Estremera, Elena Rigo, Guillermo J. Amengual, Manuel Rodríguez, José Luis Ribes, Isabel Gomila, Félix Grases, Marta González-Freire, Lluís Masmiquel

**Affiliations:** 1 Vascular and Metabolic Diseases Research Group, Endocrinology Department, Son Llàtzer University Hospital, Health Research Institute of the Balearic Islands (IdISBa), 07120 Palma de Mallorca, Spain; antelm.pujol@gmail.com (A.P.); lmasmiquel@gmail.com (L.M.); 2 Laboratory of Renal Lithiasis Research, University of Balearic Islands, Research Institute of Heath Science (IUNICS), Health Research Institute of Balearic Islands (IdISBa), 07120 Palma de Mallorca, Spain; 3 CIBER Fisiopatología de la Obesidad y Nutrición (CIBERObn). Instituto de Salud Carlos III, 28029 Madrid, Spain; 4 Neuropsychology and Cognition, Department of Psychology, Research Institute of Heath Science (IUNICS), University of Balearic Islands, Health Research Institute of Balearic Islands (IdISBa), 07120 Palma de Mallorca, Spain; 5 Neurology Department, Son Llàtzer University Hospital, 07198 Palma de Mallorca, Spain; 6 Neuroradiology Department, Son Llàtzer University Hospital, 07198 Palma de Mallorca, Spain; 7 Balearic Research Group on Genetic Cardiopathies, Sudden Death, and TTR Amyloidosis, Health Research Institute of the Balearic Islands (IdISBa), 07120 Palma de Mallorca, Spain; 8 Neuroopthalmology Department, Son Llàtzer University Hospital, 07198 Palma de Mallorca, Spain; 9 Clinical Analysis Department, Son Llàtzer University Hospital, 07198 Palma de Mallorca, Spain; 10 Clinical Toxicology Research Group, Health Research Institute of the Balearic Islands (IdISBa), 07120 Palma de Mallorca, Spain; 11 Translational Research in Aging and Longevity (TRIAL) Group, Health Research Institute of the Balearic Islands (IdISBa), 07120 Palma de Mallorca, Spain; martagonzalezfreire@gmail.com; 12 Faculty of Experimental Sciences, Universidad Francisco de Vitoria (UFV), 28223 Madrid, Spain

**Keywords:** type 2 diabetes mellitus, cognitive impairment, metabolic-associated fatty liver disease, liver fibrosis, cognitive performance

## Abstract

The effect of liver fibrosis on mild cognitive impairment (MCI) and dementia risk in type 2 diabetes mellitus (T2DM) patients is unclear. Therefore, we performed a prospective cross-sectional study on 219 patients with T2DM and older than 60 years to evaluate the association between liver fibrosis, liver steatosis, and cognitive impairment. The Montreal Cognitive Assessment (MoCA) was used to screen for MCI or dementia. Liver fibrosis was estimated using the non-invasive Fibrosis-4 (FIB-4) score, and liver steatosis was assessed with the hepatic steatosis index. The mean age was 71 ± 6 years, 47% were women and according to MoCA cut-off values, 53.88% had MCI and 16.43% had dementia. A moderate or high risk of advanced fibrosis was significantly higher in patients with MCI or dementia compared to those with normal cognition (*p* < 0.001). After adjusting for confounders, a FIB-4 score greater than 1.54 was associated with MCI or dementia (*p* = 0.039). Multivariate analysis identified age over 70.5 years, antiplatelet medication use, and a FIB-4 score above 1.54 as the most relevant risk factors. Liver fibrosis, but not liver steatosis, is associated with MCI or dementia in older T2DM patients, suggesting that FIB-4 score might be a simple biomarker for the detection of cognitive impairment.

## 1. Introduction

The obesity pandemic has led to an exponential increase in type 2 diabetes mellitus (T2DM) and metabolic-associated fatty liver disease (MAFLD), previously known as non-alcoholic fatty liver disease (NAFLD) [1]. The prevalence of T2DM has doubled since the 1980s [2]. Meanwhile, the prevalence of MAFLD already affects one third of the global population [3]. Both conditions share pathophysiology, resulting in a bidirectional relationship between them [1]. These pathologies increase the risk of cardiovascular diseases, cancer, cognitive impairment (CI), and dementia (DE).

DE affects 43.8 million people worldwide [4]. This condition is chronic, progressive, and irreversible. Early intervention in risk factors for DE, such as lower education, hypertension, hearing impairment, smoking, obesity, depression, physical inactivity, diabetes, low social contact, excessive alcohol consumption, traumatic brain injury, and air pollution, might prevent or delay 40% of DE cases [5]. The comorbidities of type 2 diabetes mellitus [6] and MAFLD [7] increase the risk of cognitive impairment development and the risk of progression from such impairment to dementia. Metabolic diseases not only cause vascular DE through cerebrovascular disease but also increase the risk of Alzheimer’s disease through mechanisms that are not fully understood [8].

It is hypothesized that type 2 diabetes mellitus would be a precursor to cognitive impairment [9]. Individuals with T2DM have a 1.5–2.5 times higher probability of developing DE [10]. Several authors suggest that glucotoxicity causes an increase in neuronal insulin resistance, impaired insulin signaling, a pro-inflammatory state, mitochondrial dysfunction, and vascular damage, leading to the deposition of β-amyloid and tau protein [11,12]. These pathophysiological mechanisms would explain the relationship between both pathologies. Therefore, the American Diabetes Association (ADA), in its consensus published in 2023, recommends, with level B evidence, screening for the early detection of mild cognitive impairment (MCI) or DE for adults 65 years of age or older at the initial visit, annually, and as appropriate [13]. In this document, they advocate for the use of three screening tools without preference for any of them (Mini-Mental State Examination, Mini-Cog, and the Montreal Cognitive Assessment). The presence of cognitive impairment hinders the ability to perform complex tasks and reduces metabolic control [14]. Additionally, poor metabolic control impairs cognitive health [15].

MAFLD comprises the progressive disease spectrum from simple steatosis through non-alcoholic steatohepatitis (NASH), with or without fibrosis, to cirrhosis [16]. Both HIS (Hepatic Steatosis Index) and FIB-4 (Fibrosis-4) are useful tools in assessing metabolic-associated fatty liver disease (MAFLD), providing information about the risk of having NASH and the degree of liver fibrosis, respectively. In some studies, simple hepatic steatosis has not been linked to cognitive dysfunction, so the presence of fibrosis would be needed to increase the probability of cognitive impairment [12]. Several studies associate MAFLD with cognitive impairment [7,8]. Even without the presence of cirrhosis, hepatic fibrosis would be a risk factor for the development of cognitive impairment [6]. The mechanisms linking MAFLD to CI are not fully understood. Some authors hypothesize that as intrahepatic fat accumulation progresses, damaged hepatocytes secrete an excess of pro-inflammatory cytokines [17]. This can activate microglia and increase the permeability of the blood–brain barrier (BBB) [18], resulting in the transmission of immune cells and neurotoxic factors to the brain. Neuroinflammation increases the formation of beta-amyloid plaque, and liver dysfunction would reduce beta-amyloid clearance, resulting in an increase in total beta-amyloid concentration [19,20]. These mechanisms would cause neuronal cell injury or death. Lin et al., 2024, suggest that MAFLD causally reduces global surface area and changes in the cortical structures of several brain gyri [21]. Neuroinflammation would not be the only mechanism linking MAFLD and cognitive impairment. MAFLD and T2DM share insulin resistance as a key pathophysiological feature. Insulin resistance diminishes the expression of insulin receptors in the brain, which in turn impairs neuronal plasticity, neuroprotection, neuronal growth, and energy metabolism—key functions that insulin normally supports under healthy physiological conditions [22]. Moreover, lipotoxicity characterized by the harmful accumulation of lipids in non-adipose tissues, particularly in the liver, arises from the release of free fatty acids (FFAs) from insulin-resistant adipocytes. This process triggers inflammatory pathways, cellular dysfunction, and lipoapoptosis. In the brain, the dysregulation caused by lipotoxicity affects orexin, a neuropeptide essential for cognitive functions, executive function, and learning [23]. An additional pathophysiological factor in MAFLD-related cognitive impairment is the alteration of the intestinal microbiota, known as dysbiosis. Dysbiosis is linked to increased intestinal permeability to pathogen-associated molecular patterns (PAMPs), such as lipopolysaccharides (LPS), and other bacterial products [24].

The primary purpose of our work is to study the association between liver fibrosis, liver steatosis, and cognitive impairment in a cohort of people living with T2DM aged over 60 years. For this purpose, we analyzed the basal data from the PHYND study. Briefly, the PHYND is a unicentric, randomized, double-blind, placebo controlled clinical trial designed to assess the effect of oral phytate supplementation on cognitive performance, metabolic control, brain iron deposition, and diabetic retinopathy on elderly patients with mild cognitive impairment and type 2 diabetes mellitus [25].

## 2. Materials and Methods

### 2.1. Subjects and Design

For this purpose, we performed a prospective cross-sectional study. Two hundred and nineteen consecutive patients with T2DM older than 60 years were recruited in Son Llátzer University Hospital (Balearic Islands, Spain) from March 2022 until January 2023. From these 219 patients, 3 groups were created based on cognitive status: “non-cognitive impairment (non-CI)”, “mild cognitive impairment (MCI)”, and "dementia (DE)”. To establish the groups, we relied on the cutoff points established in the same MoCA test based on race/ethnicity and years of education [26].

### 2.2. Data Recollection

#### 2.2.1. Clinical and Anthropometric Data

Patients’ clinical history was accessed via electronic medical records. During the study, anamnesis, laboratory analysis, and physical exams were gathered prospectively. Physical and anthropometric measurements were taken by competent professionals while the individuals were barefoot and dressed in light clothing. 

#### 2.2.2. Laboratory Data

Blood samples were taken in the morning (after a 12 h fast). These samples were allowed to remain at room temperature for 30 min before being centrifuged to separate the serum.

An automated analyzer (Cell-Dyn Sapphire and Architect ci16200, Abbott, Chicago, IL, USA) was used to perform hematimetric and biochemical assays. Insulin was measured using a chemiluminescent immunoassay (Advia Centaur, Siemens, Munich, Germany).

#### 2.2.3. Diagnostic Assessment

Type 2 diabetes was diagnosed if subjects presented fasting serum glucose ≥126 mg/dl (7.0 mmol/L) and/or glycated hemoglobin ≥6.5 % or were under treatment with hypoglycemia drugs [27].

Blood pressure was measured three times after 5 min of rest, while the participant was sitting quietly. The average of the second and third measurement was recorded. Patients using antihypertensive drugs as well as those with systolic blood pressure ≥140 mmHg and/or diastolic blood pressure ≥ 90 mmHg were categorized as having hypertension [28]. 

Dyslipidemia was defined as the presence of one of the following factors: LDL cholesterol levels ≥130 mg/dL, HDL cholesterol <40 mg/dL (in men) or <50 mg/dL (in women), triglycerides ≥150 mg/dL, or lipid-lowering drug treatment. The remission of dyslipidemia was considered if cholesterol and triglycerides were below the diagnostic cutoff values in the absence of lipid-lowering drugs [29].

Chronic kidney disease (CKD) is defined as kidney damage or glomerular filtration rate (GFR) <60 mL/min/1.73 m^2^ for 3 months or more, irrespective of cause [30]. Diabetic retinopathy was diagnosed due to a comprehensive evaluation by an ophthalmologist, which includes dilated slit-lamp examination including biomicroscopy with a hand-held lens (90 or 78 diopter), indirect ophthalmoscopy, and testing as appropriate that may include optical coherence tomography and fluorescein angiography [31].

#### 2.2.4. Liver Fibrosis and Steatosis Risk Assessment

The degree of liver fibrosis was estimated using the non-invasive formula of Fibrosis-4 (FIB-4) liver fibrosis score. We calculated the Fibrosis-4 (FIB-4) score for each participant as follows: FIB-4 = age (years) × AST (U/L)/[PLT(10^9^/L) × ALT1/2 (U/L)]. The FIB-4 score has been validated to have good accuracy for the non-invasive detection of liver fibrosis across multiple underlying etiologies. With B grade evidence, the ADA recommends in their 2023 consensus that “Adults with type 2 diabetes or prediabetes, particularly those with obesity or cardiometabolic risk factors/established cardiovascular disease, should be screened/risk stratified for nonalcoholic fatty liver disease with clinically significant fibrosis (defined as moderate fibrosis to cirrhosis) using a calculated fibrosis-4 index (derived from age, ALT, AST, and platelets even if they have normal liver enzymes)” [32]. FIB-4 was categorized as moderate (FIB-4 between 1.45 and 3.25) or high (FIB-4 > 3.25) risk of advanced fibrosis [33,34]. Some authors consider the specificity for advanced fibrosis to be unacceptably low in patients aged ≥65, suggesting that higher thresholds should be established in elderly patients. Nevertheless, we show that FIB-4 thresholds non-adjusted by age have a better performance to link the risk of moderate or advance hepatic fibrosis to cognitive performance (explained in detail further). 

The liver steatosis risk was assessed by the hepatic steatosis index (HSI). We calculated the hepatic steatosis index (HSI) = 8 × (ALT/AST ratio) + BMI (+2 if female; +2 if diabetes mellitus). HSI was categorized as < 0.0 (low risk of hepatic steatosis), 30–36 (intermediate risk of hepatic steatosis), and >36 (high risk of hepatic steatosis) [35].

### 2.3. Defining Cognitive Performance as an Outcome Variable

Cognitive screening was performed using the Montreal Cognitive Assessment Test (MoCA). We stratified MoCA results by race/ethnicity and education level before applying a cutoff value for the MoCA score to achieve more accurate cutoffs, as suggested by Milani et al., 2018 [26]. SAGE scores of 17 and above are suggestive of a normal condition, 15 and 16 are suggestive of an MCI condition, and 14 and below suggestive of a dementia condition [36]. 

### 2.4. Statistical Analysis 

The Kolmogorov–Smirnov or Shapiro–Wilk tests and normality graphs (histogram, Q-Q plot) were used to determine whether the variables follow a normal distribution. Continuous variables are expressed as “mean ± Standard Deviation”, “mean ± Standard Error”, or “median [interquartile range]”. Categorical variables are expressed as “frequency (percentage)”. 

For normally distributed variables, the independent samples *t*-test was used to compare variables between two groups. For more than two groups, the two-tailed ANOVA test was used to determine the *p*-value of differences, and the Bonferroni test was used as a post hoc test to evaluate differences. For non-normally distributed variables, the Kruskal–Wallis non-parametric test and the Mann–Whitney U test were used. Categorical variables were compared using the chi-square test or Fisher’s exact test to determine differences between groups. *p*-values for the trend were calculated using linear regression analysis.

ROC curves of quantitative risk factors associated with MCI or DE were performed. The optimal cutoff values of age and FIB-4 were determined by the maximum Youden index (J), defined as sensitivity + specificity − 1. Linear regression models were fitted to assess the associations between FIB-4 and MOCA subscales. Binary logistic regression models were used to identify risk factors associated with MCI or DE with normal cognitive function as the reference (odds ratio [OR] = 1). Analysis was performed using the stepwise backward method. A two-tailed *p*-value less than 0.05 was considered statistically significant. Statistical analyses were performed using SPSS 23.0 (SPSS Inc., Chicago, IL, USA). The Statistical Package for Social Sciences (SPSS) was used for statistical calculations. 

## 3. Results

### 3.1. Baseline Patient Characteristics 

Demographic and clinical characteristics of the three groups are shown in Table 1. The median age was 70.6 years (interquartile range (IQR): 66.0–75.3) and patients with MCI or DE were older than those with a normal cognitive condition (*p* < 0.05). The prevalence of MCI in our sample was 53.9% and the prevalence of DE was 16.4%. Normal cognition was reported in 29.7%. In total, 54.1% of patients were male, the median BMI was 31 (±5.9), and 196 patients (89.9%) had a duration of T2DM of 10 years or more. None of these parameters presented significant differences between groups. Patients with MCI or DE did not present more diabetic complications, such as diabetic nephropathy, atherosclerosis, or chronic kidney disease. Regarding medication, a higher percentage of patients with high MCI or DE were taking calcium antagonists and antiaggregant when compared to patients with normal cognitive function (*p* = 0.034 and *p* = 0.001, respectively).

### 3.2. Laboratory Analysis Parameters

Table 2 shows the clinical and biochemical characteristics among MoCA groups. As can be seen, GFR (calculated with MDR4IDMS formula and CKD-EPI formula) was lower for the DE group compared to the non-CI and MCI group [DE 66 (44–88); MCI 75 (55–95); non-CI: 77 (59–95) mL/min/1.73 m^2^; *p* = 0.004]. Hemoglobin and albumin levels decreased as the MoCA punctuation decreased (*p* < 0.001), whereas blood levels of urea and creatinine and the urinary albumin/creatinine ratio significantly increased with lower MoCA punctuation (*p* < 0.05). No significant difference between groups in HbA1c was reported. 

### 3.3. Fibrosis Score and Hepatoestatosis Index among MoCA Groups

**Figure 1:**
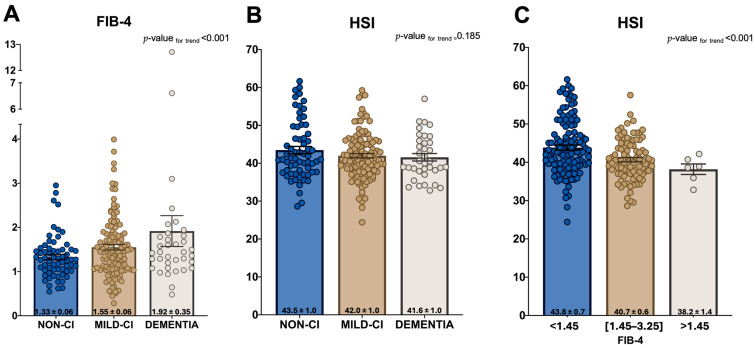
FIB-4 values among MoCA groups (**A**), HSI values among MoCA groups (**B**), and HIS values among FIB-4 groups (**C**). Values are expressed as mean ± SE. The *p*-values for trends were calculated through linear regression, treating the variables of groups as categorical (*X*-axis) and the others as continuous (*Y*-axis).

The data showed a tendency for FIB-4 values to increase as MoCA scores decreased. The median (±SE) values of FIB-4 were 1.9 (±0.35), 1.55 (±0.06), and 1.33 (±0.06) for DE, MCI, and non-CI, respectively (Figure 1A, *p*-value for trend = 0.006). However, there was no trend towards greater HSI value as MoCA punctuation decreased. The median (±SE) values of HSI were 41.6 (±1.0), 42.0 (±0.6), and 43.5 (±1.0) for DE, MCI, and non-CI, respectively (Figure 1B, *p*-value for trend = 0.185). Furthermore, there was an observed trend of higher FIB-4 values as HIS scores decreased. The median (±SE) values of HSI were 43.8 (±0.7), 40.7 (±0.6), and 38.2 (±1.4) for FIB-4 < 1.45, FIB-4 (1.45–3.25), and FIB-4 > 3.25, respectively (Figure 1C, *p*-value for trend = 0.001).

### 3.4. Fibrosis Score and MoCA Subscales

Univariate and multivariate linear regression analyses were used to investigate the association of the MoCA subscales with FIB-4 values. Table 3 shows crude and adjusted beta coefficients adjusted by age, gender, CKD, coronary disease, betablockers, calcium antagonists, and antiaggregant medication use. As observed, there is a significant correlation between elevated FIB-4 values and reduced language fluency scores in the MoCA subscales, both in adjusted and crude analyses. A significant negative association was also observed, and FIB-4 between total MoCA scores.

### 3.5. Optimal Cut-off of Age and FIB-4 Values Associated with MCI or Dementia (vs. no-CI) in Patients with T2DM

**Figure 2:**
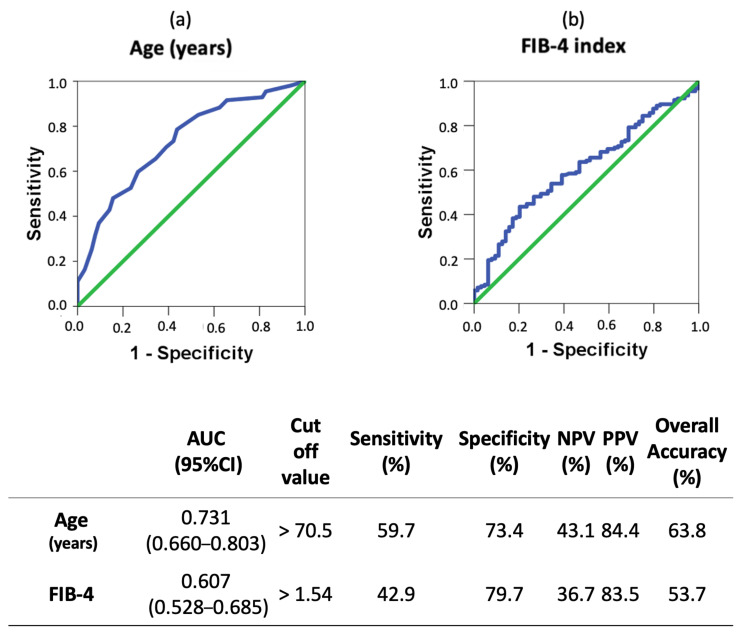
ROC curves and optimal cut-off of age and FIB-4 values associated with MCI or dementia (vs. no-CI) in patients with T2DM. The optimal cut-off values were determined by the maximum Youden index (J), defined as sensitivity + specificity − 1. The table indicates the area under the curve (AUC), sensitivity, specificity, positive predictive value (PPV), negative predictive value (NPV), and overall accuracy of the optimal cut-off values. Green line: optimal value. Blue line: obtained value.

ROC curves and optimal cut-off values were calculated for quantitative risk factors associated with moderate–severe FIB-4 value (Figure 2). As observed, FIB-4 values greater than 1.54 exhibited a sensitivity of 42.9% and specificity of 79.7%. The overall accuracy for FIB-4 above 1.54 exhibited was 53.7%. For individuals aged over 70.5 years, sensitivity and specificity were 59.7% and 73.4%, respectively. The overall accuracy for age above 70.5 reached 63.8%, surpassing that of the other analyzed variables. 

### 3.6. Risk Factors Associated with MCI or Dementia

Univariate and multivariate logistic regression analyses were used to investigate the most relevant risk factors associated with the presence of MCI or DE (vs. normal cognition). The relevant listed factors previously reported in Table 2 and Table 3 were initially included in the models before employing either the enter method (multivariate model 1) or stepwise and backward elimination (multivariate model 2) (Table 4). In the multivariate model 1, only three variables remained significantly associated with MCI or DE after adjusting for the rest of variables: age older than 70.5 years, use of antiaggregant, and FIB-4 scores higher than 1.54. The final multivariate model 2 identified age older than 70.5 (3.33 (1.68–6.61); *p* = 0.001), use of antiaggregants (3.85 (1.88–7.90); *p* < 0.001), and FIB-4 higher than 1.54 (2.19 (1.04–4.63) *p*-value = 0.039) as the most important risk factors associated with MCI or DE in T2DM. 

## 4. Discussion

Our study is the first one to report, using FIB-4 and HIS, that FIB-4 but not HSI values are negatively correlated with MoCA punctuation in elderly subjects with T2DM. The presence of a FIB-4 value higher than 1.54 is associated with a 2-fold to 3-fold higher risk for MCI or DE in elderly patients with T2DM. Seo et al., utilizing data from the 1988–1994 National Health and Nutrition Examination Survey (NHANES), which included 874 individuals diagnosed with MAFLD and 3598 healthy controls under the age of 59, found that MAFLD was linked to impaired memory and attention even after accounting for significant confounding variables [37]. However, Weinstein et al., analyzed NHANES data collected between 2011 and 2014, and contrary to expectations, an MAFLD diagnosis on its own did not correlate with decreased cognitive performance across any of the administered tests [38]. However, individuals with both MAFLD and concurrent T2DM exhibited poor cognitive performance. In another work, by Weinstein et al., while MAFLD itself did not show an independent association with cognitive dysfunction, a subset of MAFLD patients at a heightened risk of hepatic fibrosis, as indicated by the NAFLD fibrosis score (NFS), demonstrated compromised cognitive performance compared to those at lower risk [39]. In summary, the investigations mentioned fail to conclusively demonstrate an independent link between the complete range of MAFLD conditions and cognitive impairment [16]. Nevertheless, there appears to be a correlation between cognitive abilities and the severity of liver fibrosis. Those living with T2DM seem to have a higher risk of poor cognitive performance when hepatic fibrosis appears. Tools like HIS and FIB-4 are essential for assessing the risk of NASH and liver fibrosis in MAFLD. While simple hepatic steatosis has not consistently been linked to cognitive dysfunction, fibrosis appears to increase the likelihood of cognitive impairment [12]. The exact mechanisms are not fully understood, but it is hypothesized that the accumulation of intrahepatic fat leads to the secretion of pro-inflammatory cytokines, activating microglia and increasing blood–brain barrier permeability [17,18]. This can result in the transmission of immune cells and neurotoxic factors to the brain, increasing beta-amyloid formation and reducing its clearance, ultimately leading to neuronal injury or death [19,20]. Furthermore, MAFLD and T2DM share insulin resistance as a key pathophysiological feature, which diminishes insulin receptor expression in the brain, impairing critical functions like neuronal plasticity and energy metabolism [22]. Lipotoxicity can further exacerbate cognitive dysfunction through the release of free fatty acids and the subsequent triggering of inflammatory pathways [23]. Additionally, dysbiosis is linked to increased intestinal permeability and the entry of bacterial products into circulation, further contributing to cognitive decline [24].

It is worth underlining that we developed ROC curves and searched for the optimal cut-off values associated with MCI or DE. Remarkably, FIB-4 shows good accuracy, for example with age being the strongest predictor of risk to develop MCI or DE. All these data suggest that FIB-4 (with an optimal cut-off value of 1.54) could be a useful marker to identify those elderly patients living with T2DM with a higher risk of developing MCI or DE. However, McPherson et al., established higher cut-off values to exclude advanced fibrosis patients using the age-specific cut-offs [33]. They show that age has a significant effect on the performance of simple non-invasive fibrosis scores in excluding advanced fibrosis. There was a significant decrease in specificity for advanced fibrosis in older patients (≥65 years), resulting in a high false positive rate for advanced fibrosis [33]. Because of this, they established higher cut-off values for the FIB-4 score with punctuation under 2 excluding fibrosis, with scores between 2 and 2.67 requiring further investigation and with scores higher than 2.67 showing a likely diagnosis of fibrosis [33]. Nevertheless, the prevalence of MAFLD in elderly patients with T2DM is 56.9%, conferring a higher risk of developing hepatic fibrosis [40]. We should consider that elderly patients with T2DM may not benefit from McPherson-proposed cut-off values for cognitive impairment prediction. It would therefore be worth developing further prospective studies to draw definitive conclusions about the application of FIB-4 in elderly patients with T2DM.

Our results support the notion that a higher FIB-4 score is associated with a lower cognitive performance, especially in language fluency. This contradicts previous results. Executive functioning, closely linked to metabolic conditions like obesity and diabetes, may be more sensitively detected by the MoCA due to its focus on frontal tasks [8,41]. Kang et al., found that MAFLD was correlated with reduced visuospatial and executive function [8], while Seo et al., observed decreased performance in specific cognitive tests among MAFLD patients in the NHANES study [37]. Other studies suggest that MAFLD may affect cognitive performance through region-specific mechanisms rather than diffuse dysfunction [38], particularly impacting language domains [42,43]. Further research using sensitive and valid cognitive tools is needed to confirm these associations.

Secondly, we report a higher-than-expected prevalence of MCI and DE. You et al., in a systematic review and meta-analysis, demonstrated in a subgroup analysis that the prevalence of MCI in patients older than 60 years was 44.3% [44]. In Spain, a nationwide discharge database selecting T2DM patients aged 60 years or older admitted to Spanish hospitals from 2011 to 2020 found a prevalence of dementia of 8.31% [45]. We report a prevalence of MCI of 53.88% and a prevalence of DE of 16.43%. Simo et al., found results similar to those reported in our study, emphasizing the importance of cognitive impairment screening in patients with type 2 diabetes [46]. Obviously, our sample is small and it is a cross-sectional study from a single medical center; therefore, our findings should be interpreted with caution. However, if cognitive function screening became routine, we might realize that cognitive decline in individuals with type 2 diabetes is currently underdiagnosed. 

Our results indicate that age is the strongest associated factor for developing MCI or dementia in patients with T2DM. Being older than 70 years is associated with a 3-fold to 4-fold higher risk for MCI or DE in elderly patients with T2DM. Similar results have been found in other studies; in fact, aging is the primary risk factor for the development of prevalent neurological disorders [47]. Concerning other factors associated with lower cognitive performance, in our study, the median BMI was 31 (grade I obesity) and higher or lower BMI levels were not associated with cognitive performance. Evidence from longitudinal population-based studies indicates that living with obesity in midlife is linked to a higher risk of dementia later in life [48]. Some studies suggest an “obesity paradox” in late-life, where obesity appears to protect against cognitive decline and dementia; others refute this notion [49]. On the other hand, some studies have demonstrated a negative impact of high BMI in later life on cognition and the risk of dementia [50]. The current study did not find any notable correlation between the duration of T2DM and cognitive impairment. This finding contradicts the results of numerous prior studies that have explored this association. Several studies revealed that individuals diagnosed with T2DM for over five years exhibit poorer cognitive performance compared to those recently diagnosed [51,52]. We should keep in mind that most of the patients in this study have been living with T2DM for more than 10 years. Moreover, our results show that patients with MCI or DE did not present more diabetic complications, such as diabetic nephropathy, atherosclerosis, and chronic kidney disease. Legdeur et al., found, in 442,428 individuals without dementia aged ≥65 years from the longitudinal primary care Integrated Primary Care Information (IPCI) database, that vascular disorders are no longer a risk factor for dementia at older ages (>65 years) [53].

Thirdly, in our study, the use of antiaggregants is associated with a 3- to 4-fold higher risk for MCI or DE in elderly patients with T2DM. We report a higher use of antiplatelets, and especially low-dose aspirin, in those with lower cognitive performance. Some authors hypothesized that low-dose aspirin could potentially safeguard against dementia and cognitive decline via two primary mechanisms [54,55]: by influencing Alzheimer’s disease-related pathology and by reducing vascular events. Li et al., showed that in cohort studies, it was observed that the use of low-dose aspirin was associated with a potential decrease in the occurrence of dementia, a finding not corroborated by randomized controlled trials (RCTs). However, the available evidence was deemed insufficient to comprehensively assess the impact of aspirin on cognitive function and dementia [55]. Recently, Cloud et al., revealed a notable rise in intracranial bleeding among individuals taking daily low-dose aspirin, yet their findings did not show a significant decrease in ischemic stroke incidence. Therefore, low-dose aspirin is not recommended in primary prevention. These results could be explained by reverse causality or by a prescription bias effect. 

Also, a higher percentage of patients with high MCI or DE were taking calcium antagonists compared to patients with normal cognition. Hussain et al., in a meta-analysis based on ten observational studies with a total of 75,239 patients, found that elderly hypertensive patients who took calcium antagonists experienced a significant reduction of 30% in the risk of developing dementia [56]. In vitro data suggest that the use of calcium antagonists may minimize the formation of amyloid beta peptide [57]. Our results in these domains could be explained by reverse causality or by the prescription bias effect.

Chronic kidney disease (CKD) is one of the strongest risk factors for mild cognitive impairment and dementia [58], and early cognitive impairment also emerges in the initial stages of CKD and progresses alongside the decline in kidney function [59]. We report a lower glomerular filtration rate (GFR) (calculated with MDR4IDMS formula and CKD-EPI formula) for the DE group compared to the non-CI and MCI group. Although the exact mechanism between CKD and CI is not fully understood, systemic hypertension, arteriosclerosis, uremic toxin-related pathways, and the increase in low-grade inflammation and oxidative stress would be key factors in the brain–kidney relationship [58]. In this study, we report that the urinary albumin/creatinine ratio significantly increases with lower MoCA punctuation. It has been reported that albuminuria is independently associated with incident cognitive impairment. In fact, urinary albumin/creatinine ratios of 30–299 and ≥300 mg/g were associated independently with 31% and 57% higher risks of cognitive impairment, respectively, relative to individuals with albumin/creatinine ratios lower than 10 mg/g [60]. Albuminuria is strongly correlated with the onset of cognitive impairment, especially at higher estimated glomerular filtration rates (eGFR) [60]. Creatinine-based GFR estimates, like the CKD-EPI equation, often overestimate GFR in individuals with low muscle mass, potentially misclassifying kidney function in malnourished individuals at higher risk of cognitive decline. Additionally, albuminuria with a high eGFR may indicate glomerular hyperfiltration, linked to systemic microvascular abnormalities that accelerate kidney and cognitive decline [60,61]. We also observed that decreases in hemoglobin and albumin levels correlated with lower MoCA scores, reflecting the impact of anemia and malnutrition in CKD on brain oxygenation and metabolism, exacerbating cognitive impairment. Higher blood urea levels were also associated with lower cognitive performance [58].

Lastly, we report a trend of higher FIB-4 values as HIS scores decreased. In terms of pathogenesis, toxic, metabolic, or viral diseases result in hepatocyte damage and immune cell infiltration, which triggers the activation of hepatic stellate cells (HSCs) to transform into collagen-producing myofibroblasts [62]. The substitution of liver tissue by fibrotic tissue is expected if the hepatic injury persists. We are the first ones to show HIS score decreases as FIB-4 values, which could represent a marker of the progression of MAFLD to NASH.

Our study has several limitations. First, the sample is small, and it is a cross-sectional study from a single medical center; therefore, the findings presented here should be interpreted with caution. Even though we found that age, FIB-4 score, calcium antagonist use, antiaggregant use, hemoglobin, albumin blood levels of urea, creatinine, and albumin/creatinine ratio were associated with lower cognitive function, this does not prove causality. We utilized the FIB-4 index to assess the likelihood of liver fibrosis, acknowledging its potential for reduced specificity in certain scenarios. However, for the most accurate diagnosis of liver fibrosis, liver biopsy remains the gold-standard diagnostic tool. By including patients with cerebrovascular disease, we acknowledge the possibility of cognitive impairment resulting from these events. However, our decision to not exclude these patients was intended to enhance the ecological validity of our findings. It is important to note that the influence of cerebrovascular disease on cognitive outcomes should be considered when interpreting our results. Future studies may benefit from stratifying patients based on the presence or absence of cerebrovascular disease to further clarify its impact on cognitive function in T2DM populations.

Prospective multicentric longitudinal studies are needed to fully clarify how hepatic fibrosis contributes to the genesis and progression of MCI to DE and to assess the possibility that FIB-4 score may serve as biomarker to predict MCI. 

## 5. Conclusions

We demonstrate that liver fibrosis, but not liver steatosis, is associated with MCI or dementia in older patients with T2DM. These findings have potentially important clinical implications because FIB-4 score might be a simple biomarker for the detection of cognitive impairment. A FIB-4 score greater than 1.54 in individuals older than 65 years could be a useful tool for identifying individuals at high risk of cognitive impairment. Antiaggregant use and age are also relevant factors associated with a worse cognitive performance. Nevertheless, prospective studies are needed to establish the time sequence in this relationship and clinically relevant findings.

## Figures and Tables

**Table 1:** Clinical characteristics of patients among MoCA groups. Each value is given as mean ± standard deviation or frequency (percentage). The significance of differences between groups was determined using a one-way ANOVA or Kruskal–Wallis test for quantitative data and a chi-square test for qualitative data.

	All Patients	Normal Cognitive Function	Mild Cognitive Impairment	Dementia	*p*-Value
	(*n* = 219)	(*n* = 64)	(*n* = 118)	(*n* = 36)	
**Anthropometric Measures**						
Age (years)	70.6 ± 6.2	67.0 ± 5.2	71.4 ± 6.2	73.9 ± 4.7	<0.001	
Gender (male)	118 (54.1%)	35 (54.7%)	65 (55.1%)	18 (50.0%)	0.861	
BMI (kg/m^2^)	31.0 ± 5.9	31.6 ± 6.7	30.9 ± 5.6	30.4 ± 5.1	0.558	
Waist circumference (cm)	110 ± 16	112 ± 17	109 ± 15	107 ± 13	0.502	
Systolic blood pressure (mmHg)	144 ± 19	139 ± 18	145 ± 20	144 ± 20	0.162	
Diastolic blood pressure (mmHg)	79 ± 11	78 ± 12	80 ± 11	77 ± 10	0.448	
Pulse pressure (mmHg)	79 ± 10	78.6 ± 7.8	79 ± 10	80 ± 13	0.946	
**Comorbidities, Toxics, and Clinical History**						
Time from diagnosis DM2						
Less than 5 years	8 (3.7%)	3 (4.7%)	4 (3.4%)	1 (2.8%)	0.794	
Between 5 and 10 years	14 (6.4%)	6 (9.4%)	6 (5.1%)	2 (5.6%)		
More than 10 years	196 (89.9%)	55 (85.9%)	108 (91.5%)	33 (91.7%)		
Diabetic retinopathy	18 (8.3%)	3 (4.7%)	12 (10.2%)	3 (8.3%)	0.439	
Diabetic nephropathy	38 (17.4%)	6 (9.4%)	23 (19.5%)	9 (25.0%)	0.097	
Intermittent claudication	3 (1.4%)	2 (3.1%)	1 (0.8%)	0 (0.0%)	0.335	
Cerebrovascular accident	10 (4.6%)	4 (6.3%)	6 (5.1%)	0 (0.0%)	0.330	
Polyneuropathy	19 (8.7%)	7 (10.9%)	11 (9.3%)	1 (2.8%)	0.359	
Diabetic foot	5 (2.3%)	0 (0.0%)	4 (3.4%)	1 (2.8%)	0.337	
Intermittent claudication	4 (1.8%)	1 (1.6%)	3 (2.5%)	0 (0.0%)	0.598	
Atherosclerosis	200 (91.7%)	60 (93.8%)	105 (89.0%)	35 (97.2%)	0.288	
Hypertension	189 (86.7%)	54 (84.4%)	103 (87.3%)	32 (88.9%)	0.785	
Smoking	17 (7.8%)	7 (10.9%)	9 (7.6%)	1 (2.8%)	0.584	
Alcohol	12 (5.5%)	4 (6.3%)	6 (5.1%)	2 (5.6%)	0.637	
Prior cardiovascular disease	1 (0.5%)	1 (1.6%)	0 (0.0%)	0 (0.0%)	0.072	
Chronic kidney disease (CKD)	41 (18.8%)	8 (12.5%)	20 (16.9%)	13 (36.1%)	0.011	
2 (eGFR 89–60 mL/min/1.73 m^2^)	87 (39.9%)	26 (40.6%)	48 (40.7%)	13 (36.1%)		
3a (eGFR 59–45 mL/min/1.73 m^2^)	14 (6.4%)	3 (4.7%)	9 (7.6%)	2 (5.6%)		
3b (eGFR 44–30 mL/min/1.73 m^2^)	20 (9.2%)	4 (6.3%)	9 (7.6%)	7 (19.4%)		
4 (eGFR 29–15 mL/min/1.73 m^2^)	0 (0.0%)	4 (3.4%)	2 (5.6%)	6 (2.8%)		
**Medication Use**						
Angiotensin-converting enzyme inhibitors/angiotensin II receptor-blocking agents	177 (81.2%)	54 (84.4%)	96 (81.4%)	27 (75.0%)	0.514	
Beta-blockers	81 (37.2%)	17 (26.6%)	46 (39.0%)	18 (50.0%)	0.055	
Calcium antagonists	61 (28.0%)	13 (20.3%)	32 (27.1%)	16 (44.4%)	0.034	
Statins	187 (85.8%)	53 (82.8%)	102 (86.4%)	32 (88.9%)	0.674	
25-hydroxyvitamin D	90 (41.3%)	20 (31.3%)	53 (44.9%)	17 (47.2%)	0.148	
Calcium supplements	15 (6.9%)	2 (3.1%)	10 (8.5%)	3 (8.3%)	0.369	
Antiplatelets	90 (41.3%)	13 (20.3%)	59 (50.0%)	18 (50.0%)	<0.001	
Insulin	98 (45.0%)	33 (51.6%)	47 (39.8%)	18 (50.0%)	0.253	
Oral antidiabetic medication	211 (96.8%)	64 (100%)	114 (96.6%)	33 (91.7%)	0.075	
Uric medication (allopurinol or febuxostat))	35 (16.1%)	8 (12.5%)	20 (16.9%)	7 (19.4%)	0.614	
Thiazides	65 (29.8%)	19 (29.7%)	36 (30.5%)	10 (27.8%)	0.925	
Furosemide	28 (12.8%)	10 (15.6%)	15 (12.7%)	3 (8.3%)	0.577	
Metformin	166 (76.1%)	55 (85.9%)	85 (72.0%)	26 (72.2%)	0.092	
Semaglutide	50 (22.9%)	19 (29.7%)	27 (22.9%)	4 (11.1%)	0.105	
Liraglutide	14 (6.5%)	6 (9.4%)	6 (5.1%)	2 (5.6%)	0.524	
Dulaglutide	36 (16.8%)	8 (12.5%)	21 (17.8%)	7 (7 19.4%)	0.573	
Dapaglifozine	35 (16.1%)	8 (12.5%)	20 (16.9%)	7 (19.4%)	0.614	
Empaglifozine	65 (29.8%)	19 (29.7%)	36 (30.5%)	10 (27.8%)	0.925	
Pioglitazone	15 (6.9%)	2 (3.1%)	10 (8.5%)	3 (8.3%)	0.369	

**Table 2:** Laboratory parameters among MoCA groups. Each value is given as the mean ± standard deviation or median (interquartile range). The *p*-values for trends were calculated using linear regression, treating MoCA as categorical variables and laboratory variables as continuous data.

	All Patients (*n* = 219)	Normal Cognitive Function (*n* = 64)	Mild Cognitive Impairment (*n* = 118)	Dementia (*n* = 36)	*p*-Value for Trend
Glomerular filter MDR4IDMS (mL/min/sup)	74 ± 20	76 ± 20	75 ± 20	66 ± 22	0.013
Glomerular filter CKD-EPI (mL/min/1.73 m^2^)	74 ± 20	77 ± 18	75 ± 20	66 ± 22	0.004
Leukocytes (×10^9^/L)	7.7 ± 2.2	7.7 ± 1.9	7.6 ± 2.4	7.7 ± 2.3	0.954
Hemoglobin (g/dL)	14.0 ± 1.6	14.4 ± 1.5	14.1 ± 1.7	13.3 ± 1.6	0.001
Glucose (mg/dL)	144 ± 50	146 ± 45	141 ± 46	154 ± 69	0.440
Urea (mg/dL)	44 ± 19	43 ± 18	42 ± 17	52 ± 24	0.035
Creatinine (mg/dL)	0.96 ± 0.35	0.92 ± 0.28	0.95 ± 0.37	1.07 ± 0.39	0.042
Urate (mg/dL)	5.3 ± 1.7	5.6 ± 1.8	5.2 ± 1.7	5.2 ± 1.7	0.328
Sodium (mEq/L)	140.3 ± 2.1	140.4 ± 2.1	140.4 ± 2.1	139.9 ± 2.1	0.230
Potassium (mEq/L)	4.62 ± 0.48	4.59 ± 0.44	4.61 ± 0.45	4.73 ± 0.62	0.160
Chloride (mEq/L)	103.9 ± 3.0	104.0 ± 2.8	103.9 ± 2.9	103.7 ± 3.7	0.684
Calcium (mg/dL)	9.48 ± 0.44	9.47 ± 0.42	9.52 ± 0.43	9.37 ± 0.46	0.214
Phosphate (mg/dL)	3.48 ± 0.62	3.57 ± 0.39	3.41 ± 0.64	3.53 ± 0.86	0.887
Total cholesterol (mg/dL)	154 ± 39	153 ± 34	153 ± 41	159 ± 41	0.491
HDL cholesterol (mg/dL)	43 ± 11	43 ± 8	43 ± 11	43 ± 13	0.783
LDL cholesterol (mg/dL)	80 ± 30	80 ± 27	81 ± 33	82 ± 28	0.770
Triglycerides (mg/dL)	151 ± 85	154 ± 88	149 ± 88	153 ± 67	1.000
Albumin (g/dL)	4.38 ± 0.38	4.47 ± 0.37	4.37 ± 0.38	4.25 ± 0.39	0.004
Phosphatase alkaline (U/L)	76 ± 30	75 ± 28	75 ± 27	80 ± 44	0.401
HbA1c_NGSPDCCT (%)	7.4 ± 1.3	7.5 ± 1.3	7.3 ± 1.1	7.7 ± 1.8	0.488
25-hydroxyvitamin D3 (ng/mL)	26 ± 15	27 ± 13	25 ± 14	28 ± 19	0.692
Creatinine urine (mg/dL)	77 ± 43	72 ± 34	76 ± 42	87 ± 59	0.179
Urinary albumin/creatinine ratio (mg/g)	60 ± 146	27 ± 32	54 ± 95	132 ± 299	0.020
	13.8 [8.5–34.6]	13.1 [10.2–33.2]	14.2 [8.2–78.3]	13.7 [7.4–42.5]	
Microalbuminurie (mg/dL)	4.7 ± 14.7	2.5 ± 3.3	5.3 ± 18.9	6.8 ± 12.1	0.280
Parathyroid hormone (pg/dL)	147.9 ± 88.0	133.1 ± 66.5	149.9 ± 104.7	163.7 ± 17.9	0.652
Fibrinogen (mg/dL)	481.9 ± 84.4	497.4 ± 73.7	475.1 ± 91.9	474.6 ± 93.1	0.648
Transferrin (mg/dL)	287.2 ± 47.4	295.4 ± 48.3	283.8 ± 46.3	281.2 ± 49.6	0.310
C-Reactive protein (mg/dL)	0.88 ± 5.37	0.46 ± 0.39	1.24 ± 7.28	0.48 ± 0.44	0.978
Platelets (10^9^/L)	231.6 ± 92.8	233.5 ± 63.5	233.0 ± 110.1	223.7 ± 74.3	0.607
AST (UI/L)	20.1 ± 9.8	20.0 ± 6.9	20.0 ± 10.7	20.2 ± 11.3	0.935
ALT (UI/L)	21.1 ± 11.2	22.8 ± 11.7	20.2 ± 9.6	20.8 ± 14.7	0.439
Red cell distribution width (%)	14.0 ± 1.4	14.1 ± 1.5	14.0 ± 1.4	13.9 ± 1.2	0.435
Lymphocytes (%)	30.4 ± 9.1	30.0 ± 8.7	30.3 ± 9.0	31.5 ± 10.3	0.430
Mean cell volume (fL)	90.7 ± 7.3	91.0 ± 7.0	90.9 ± 7.2	89.8 ± 8.2	0.431
White blood cell count (1000 cells/UL)	7.7 ± 2.2	7.7 ± 1.9	7.6 ± 2.4	7.7 ± 2.3	0.954

**Table 3:** Crude and adjusted beta coefficients from linear regression showing associations of the MoCA subscales with FIB-4 values.

	Crude β Coefficient	95% CI for Crude β	*p*-Value	Adjusted β Coefficient *	95% CI for Adj. β	*p*-Value
Visuospatial/executive function	−0.103	(−0.236–0.031)	0.130	−0.066	(−0.204–−0.970)	0.343
Naming	−0.082	(−0.215–0.052)	0.229	−0.120	(−0.255–−0.375)	0.080
Attention	−0.033	(−0.167–0.101)	0.633	−0.059	(−0.201–−0.943)	0.415
Language fluency	−0.214	(−0.345–−0.083)	0.001	−0.174	(−0.318–0.973)	0.018
Abstraction	−0.115	(−0.248–0.018)	0.090	−0.133	(−0.267–−0.063)	0.054
Memory	−0.066	(−0.200–0.067)	0.329	−0.011	(−0.149–−0.278)	0.879
Orientation	0.055	(−0.079–0.189)	0.416	0.054	(−0.079–2.230)	0.423
Total MOCA	−0.151	(−0.283–−0.018)	0.026	−0.154	(−0.299–0.234)	0.038

* Adjusted by age, gender, CKD, coronary disease, betablockers, calcium antagonist, and antiaggregant medication.

**Table 4:** Crude and adjusted models of risk factors associated with MCI or dementia in patients with T2DM. Multivariate analysis was performed using the stepwise backward method. Crude and adjusted odds ratios (ORs) are indicated in the table. Multivariate model 1 includes all analyzed variables using the “enter” procedure, while Model 2 indicates only significant variables through a stepwise-backward procedure. A comparison of the expected and observed frequencies using the Hosmer–Lemeshow goodness-of-fit test (*p*-value = 0.863) and an ROC curve (AUC = 0.759; *p* < 0.001) indicated a good fit for model 2.

	Univariate Analysis			Multivariate Model 1			Multivariate Model 2		
	Crude OR	(95% C.I. for Crude OR)	*p*-Value	Adj OR	(95% C.I. for Adj. OR)	*p*-Value	Adj OR	(95% C.I.f or Adj. OR)	*p*-Value
Age > 70.5 years	4.102	(2.160–7.791)	<0.001	2.850	(1.378–5.894)	0.005	3.328	(1.675–6.612)	0.001
Gender (female)	1.016	(0.758–1.362)	0.915	1.094	(0.549–2.178)	0.798			
CKD (yes vs. no)	1.909	(0.828–4.399)	0.129	1.135	(0.427–3.019)	0.800			
Coronary disease (yes vs. no)	2.293	(1.003–5.240)	0.049	1.214	(0.401–3.673)	0.731			
Betablockers (yes vs. no)	1.966	(1.036–3.731)	0.039	1.386	(0.647–2.970)	0.402			
Antiaggregants (yes vs. no)	3.923	(1.976–7.790)	0.000	3.920	(1.751–8.775)	0.001	3.859	(1.885–7.901)	<0.001
Calcium antagonists (yes vs. no)	1.776	(0.884–3.570)	0.107	1.535	(0.689–3.420)	0.295			
FIB-4 > 1.54	2.942	(1.479–5.852)	0.002	2.365	(1.102–5.077)	0.027	2.195	(1.040–4.631)	0.039

## Data Availability

The data presented in this study are available on request from the corresponding author due to privacy reasons.
